# Nursing response and COVID-19 mortality in rural municipalities in southern Brazil

**DOI:** 10.1590/0034-7167-2024-0332

**Published:** 2025-11-07

**Authors:** Lucia Helena Donini Souto, Janaína Barbieri, Nikole Martins de Figueiredo, Angela de Oliveira Carneiro, Adriano Maia dos Santos, Adriana Roese Ramos, Deise Lisboa Riquinho

**Affiliations:** I Universidade Federal do Rio Grande do Sul. Porto Alegre, Rio Grande do Sul, Brazil.; II Universidade Federal do Vale do São Francisco. Petrolina, Pernambuco, Brazil.; III Universidade Federal da Bahia. Salvador, Bahia, Brazil.

**Keywords:** Primary Health Care, Nursing, COVID-19, Mortality, Rural Health., Atención Primaria de Salud, Enfermería, COVID-19, Mortalidad, Salud Rural.

## Abstract

**Objectives:**

to explore how the COVID-19 pandemic was addressed through prevention, health promotion, adaptation, and the reorganization of Primary Health Care services in two rural municipalities in southern Brazil with differing mortality rates.

**Methods:**

this qualitative multiple case study was conducted through semi-structured interviews with 20 participants-including Primary Health Care professionals and key stakeholders-and supported by documentary data. Three core categories emerged: “Infrastructure, equipment, and access,” “Nurse-led care coordination: monitoring and isolation,” and “Medications and vaccines: organization and distribution.”

**Results:**

health teams restructured care flows, enhanced patient monitoring, secured equipment, and repurposed physical spaces to reduce viral transmission. While strategies to address the disease were largely similar, political disagreements and the use of the so-called “COVID Kit” marked critical differences.

**Final Considerations:**

economic, political, and cultural factors may influence variations in COVID-19 mortality across rural settings.

## RESUMEN

**Objetivos:**

comprender la respuesta a la COVID-19 en términos de prevención, promoción, adaptación y reorganización de la Atención Primaria de Salud (APS) en dos municipios rurales del sur de Brasil con diferentes tasas de mortalidad.

**Método:**

estudio cualitativo de tipo estudio de casos múltiples, basado en entrevistas semiestructuradas a 20 participantes (profesionales de la APS y actores clave), junto con análisis de información documental. Emergieron tres categorías: “Equipamiento, infraestructura y acceso”; “Coordinación del cuidado por parte del personal de enfermería: monitoreo y aislamiento”; y “Medicamentos y vacunas: organización y distribución”.

**Resultados:**

los equipos reorganizaron flujos de atención, seguimiento de pacientes, adquisición de insumos y redistribución de espacios físicos para disminuir la transmisión viral. Las respuestas fueron similares, aunque se observaron divergencias en el ámbito político y en el uso del “Kit COVID”.

**Consideraciones Finales:**

factores económicos, políticos y culturales pueden influir en las diferencias de tasas de mortalidad por COVID-19.

## INTRODUCTION

Rural communities face persistent challenges, such as a shortage of healthcare professionals and significant difficulties in accessing secondary and tertiary services, often due to long distances and a lack of transportation options. The COVID-19 pandemic intensified these longstanding issues. As the coordinating level and main point of entry into Brazil’s Unified Health System (*Sistema Único de Saúde* - SUS), Primary Health Care (PHC) was strained to meet the demands such as managing and referring cases during the pandemic, particularly in rural areas, where it is often the only available service^(1,2)^.

Initially, COVID-19 cases and deaths were concentrated in urban areas. Over time, however, the disease spread widely to rural regions. Despite this shift, few studies have examined the pandemic’s impact in those settings.

To fulfill their roles effectively, professionals working within Brazil’s Family Health Strategy (*Estratégia de Saúde da Família* - ESF) in rural communities faced numerous obstacles-ranging from geographic isolation and socioeconomic constraints to informational barriers. A core tenet of the ESF model is recognizing each patient’s social and cultural context and tailoring care to the specific characteristics of their territory. This place-based approach supports comprehensive care, reinforces public health surveillance to protect community well-being, promotes prevention and control of health risks, and fosters ongoing health promotion efforts^(3,4)^.

In the state of Rio Grande do Sul (RS), crowding in urban centers played a key role in accelerating transmission. Socioeconomic factors were also decisive-whether through the export of primary goods or the concentration of labor-intensive industries such as meatpacking plants. These facilities were at the center of COVID-19 outbreaks in regional hubs like Passo Fundo and Lajeado. The high density of agribusiness operations and the daily mobility of workers also led to the virus spreading across smaller surrounding municipalities^(5,6)^.

Within RS, municipalities with greater daily commuting and higher population density showed the highest crude mortality rates. One study found a higher concentration of confirmed COVID-19 cases in areas marked by urban deprivation, including households with no income, no access to clean water or sanitation, and severe overcrowding^(7)^. Another study linked political partisanship to COVID-19 mortality, reporting that municipalities where Jair Messias Bolsonaro-former president of Brazil and member of the Liberal Party (PL)-received the most votes in both the 2018 and 2022 elections experienced an excess of COVID-19 deaths in 2020 and 2021^(8)^.

Research focused specifically on rural municipalities conjointly COVID-19 remains scarce. Little is known about how the virus affected these populations or which local strategies were implemented to reduce transmission and prevent infection. The unique characteristics of rural territories warrant further investigation to understand both the drivers behind elevated mortality and the community level responses aimed at reducing spread and saving lives^(3)^.

Given this context, the following research question emerged: How did Primary Health Care teams respond to COVID-19 in rural municipalities with differing mortality rates?

## OBJECTIVES

To explore the response to COVID-19 in the areas of prevention, health promotion, adaptation, and the reorganization of Primary Health Care (PHC) in two rural municipalities in the state of Rio Grande do Sul, Brazil, with differing mortality rates.

## METHODS

### Ethical aspects

This study is part of a larger research project entitled “Work environment and health during the COVID-19 pandemic: absenteeism, burnout, management, and work organization among nursing professionals”, approved by the Research Ethics Committee of a public hospital in the state of Rio Grande do Sul. All participants signed an Informed Consent Form (ICF) prior to the interviews.

To ensure anonymity, the letter “P” was assigned to healthcare professionals and “S” to key stakeholders, followed by a sequential number indicating the order of interviews and the municipality (Municipality A or Municipality B). Access to the material was restricted to the research team.

### Type of study

This is a descriptive qualitative study conducted using a multiple case study design. This approach enables researchers to focus on one or more distinct cases. Although the two municipalities belong to similar regions, they reported markedly differing COVID-19 mortality rates.

As a methodology, the multiple case study is a valuable strategy for investigating phenomena in real-world contexts. It allows researchers to grasp the complexity of situations, describe and understand the full scope of each case, and compare findings across cases when appropriate. This manuscript was prepared in accordance with the COREQ checklist criteria^(9,10)^.

### Study setting

The study was conducted in two rural municipalities in the state of Rio Grande do Sul, Brazil, each with contrasting COVID-19 mortality rates. Between 2020 and 2021, Municipality A had the second-highest cumulative mortality rate per 100,000 inhabitants (661), while Municipality B reported the lowest (0)^(11)^. These municipalities were selected because they belong to neighboring health regions (not disclosed to preserve research anonymity), have predominantly rural populations (71% in each)^(12)^, and are served by Primary Health Units (PHU) staffed by Family Health Teams (eSF) and Multidisciplinary Health Teams (eMulti).

Regarding sociodemographic aspects, Municipality A had an Indigenous community, part of the population worked in meatpacking plants, and the mayor at the time was affiliated with the Progressive Party (PP). Although COVID-19 cases were reported among Indigenous individuals, no deaths were recorded. Municipality B had a higher concentration of older adults and small-scale farmers. The mayor of this municipality also belonged to the PP.

### Data source

In both municipalities, all healthcare professionals (18 in Municipality A and 23 in Municipality B) and key stakeholders-such as municipal health secretaries and regional representatives from the State Health Coordinating Offices (six potential participants)-were invited to take part in the study. Inclusion criteria required at least six months of employment prior to the onset of the pandemic. Those on medical leave or vacation during data collection were excluded. Three professionals from Municipality A were on vacation during this period.

A total of 20 participants were included: 16 PHC professionals (9 from Municipality A and 7 from Municipality B) and 4 key stakeholders (1 from Municipality A and 3 from Municipality B). Participants were selected through purposive sampling to ensure representation of different professional roles. Although other eligible professionals met the criteria, some declined to participate. One key stakeholder from Municipality A initially agreed to be interviewed but later declined participation. All interviews were included in the analysis and contributed to formulating thematic categories.

Sample size was guided by the concept of “information power”, which takes into account the study aim, sample specificity, theoretical framework, quality of dialogue, and analytical strategy^(13)^. The professionals included four nurses, four nursing technicians, six community health workers, one psychologist, and one pharmacist. The key stakeholders were nurses and a health administrator.

### Data collection and organization

Before data collection began, three nurse researchers-one with a PhD, one with a master’s degree, and one specializing in qualitative methods-met with key stakeholders via videoconference to present the study, schedule site visits, and arrange the interviews. The specialist nurse conducted semi-structured interviews between June and July 2023. Each interview was held individually during working hours according to participant availability. Interviews took place in private rooms at Primary Health Units, the municipal health department, or, in the case of regional health representatives, via an online videoconferencing platform. All interviews were audiotaped and later transcribed verbatim. Each session lasted approximately 40 minutes.

In addition to the interviews, contextual information was gathered from multiple documentary sources, including municipal websites, news articles, and internal administrative records made available by the municipalities (such as meeting minutes, official memos, local decrees, and ordinances). Using multiple sources of evidence enhanced the credibility of the multiple case study. This process-known as data triangulation-was employed to establish a chain of evidence and strengthen the accuracy of findings and interpretations.

### Data analysis

The interview data underwent thematic content analysis^(14)^, conducted in three stages: pre analysis, material exploration, and interpretation of the results. The analytical process was guided by best practices related to COVID-19 and informed by both the literature and the specific study context. NVivo software (version 14.1) was used to assist in data organization and the development of analytical categories.

The multiple case study was ultimately interpreted through narrative explanation, an approach that seeks to describe a phenomenon by establishing a logical chain of causality-that is, explaining how or why something occurred.

Three analytical categories emerged to answer the study’s central research question: “Equipment, infrastructure, and access”; “Nurse-led care coordination: monitoring and isolation”; and “Medications and vaccines: organization and distribution”.

## RESULTS

### Equipment, infrastructure, and access

In Municipality A, a dedicated area was set up to care for patients with respiratory symptoms. Home visits were also conducted, and the PHU remained open 24 hours a day. During on-call shifts, two professionals were available in case any patients required urgent care. Although PPE was initially in short supply, participants reported that supply issues had been resolved after the first few months. It was also necessary to prepare an equipped room for handling severe cases if patients were unable to obtain admission at the designated referral hospital.

Participants from Municipality B reconfigured the layout of rooms within the PHU to ensure that patients with respiratory symptoms could be treated in isolation-from when they arrived to when they left the facility. Despite using PPE, limited physical space and small consultation rooms led to frequent exposure among staff members.

*We tried to set up a dedicated room for care, where patients with symptoms wouldn’t have so much contact with others* [...]. (A1 - Municipality B)

*It took a while to arrive. There were times when we had it, and times when we didn’t* [...]. *Everyone struggled with the PPE situation. Sometimes we only had that one batch-especially in the beginning, when everyone needed it.* (P1 - Municipality A)

In Municipality B, a specific care protocol was created for patients with respiratory symptoms. It included medication guidelines, three assigned professionals for care delivery, testing for flu-like symptoms, and daily monitoring of those who tested positive for COVID-19. Follow-up was done through daily phone calls, especially for patients experiencing more severe symptoms such as fever. When necessary, home visits were carried out to assess vital signs and refer patients to the hospital. According to participants, the Regional Health Coordinating Office shared technical guidelines issued by the State Health Department, and this coordination was seen as a contributing factor to the absence of COVID-related deaths in the municipality.

In both municipalities, routine care at the PHUs was scaled back to focus on patients with respiratory symptoms and emergency cases. One key stakeholder from Municipality B noted that home visits were conducted for testing, medication administration, and the care of COVID-19 patients as a strategy to reduce foot traffic at the facility. The nursing team managed patient monitoring.

*I think that was really important-to have that level of control. And here, we were able to call and keep good track of patients* [...]. (A1 - Municipality B)

*That monitoring done by the nursing team* [...] *they used a spreadsheet. They knew how many had COVID-19, how many were in isolation, and the condition of each patient.* (P1 - Municipality A)

### Nurse-led care coordination: monitoring and isolation

Participants from Municipality A reported that service coordination was led by nurses, who also participated in training and continuing education activities already in place. At that point in the pandemic, however, the priority shifted toward managing severe cases-especially patients with respiratory symptoms and risk of cardiopulmonary arrest-as well as proper PPE use and protocols. As such, nurses focused primarily on preparing for these critical scenarios.

Likewise, in Municipality B, participants described that nurses were responsible for coordinating care delivery throughout the pandemic. Triage was conducted at the PHU entrance, prioritizing patients with flu-like symptoms and those requiring urgent or emergency care. Due to increased demand, service hours were sometimes extended. Participants also pointed to the need for greater recognition of nurses and nursing technicians-particularly regarding compensation and the establishment of a national minimum wage for nurses, which had not yet been approved at the time.

*The nurse always guided us on what needed to be done, organized the home visits-but always carefully, with concern for both the community and our team, because of the risk of exposure. We have families too, so we had to be cautious.* (P2 - Municipality A)

Home visits were modified during the pandemic: they were conducted outdoors in well ventilated areas whenever possible. Group activities for older adults, people with noncommunicable chronic diseases (NCDs), and pregnant individuals were suspended to prevent gatherings.

*We didn’t go inside the house-we stayed outside, where there was airflow and better ventilation.* (P2 - Municipality B)

*We conducted home visits daily, every single day. And if the patient needed something, they could call the PHU, which had a team available 24 hours a day. We were basically on call. There’s a night shift here, but during on-call duty there would be a nursing technician and a nurse.* (P6 - Municipality A)

In Municipality A, home visits were carried out with COVID-19 patients to assess vital signs and provide daily follow-up. When needed, the nursing and medical teams conducted these visits together, as many patients were reluctant to go to the hospital. In such cases, the entire team worked to engage patients, offering guidance and encouraging them to seek care at the PHU, especially when their condition was worsening and there was risk of death. According to participants, some patients delayed going to the PHU-or refused altogether-out of fear of intubation or dying in the hospital.

*I made a visit, and the patient was in really bad shape. When I got there, it was clear they were very sick. I advised them to go to the PHU for medication and, if needed, to the hospital-but they didn’t want to go. So I spoke with the doctor and asked them to come with me to try to convince the patient to go to the PHU* [...]. (P7 - Municipality A)

In Municipality B, participants stated that patients diagnosed with COVID-19 remained in isolation for 15 days. Although technical guidelines were later updated to shorten isolation periods, participants reported that they continued recommending extended isolation, issuing medical certificates when necessary. This approach was seen as a way to reduce transmission. Patients with respiratory symptoms were also monitored through multiple communication channels, including teleconsultations, social media, radio announcements, and the municipal website.

*Honestly, we held the line for as long as we could-even with isolation. It took a while, because when the Ministry of Health shortened the isolation from 14 to 10 days, we still kept people isolated for 14. People would ask why-since it had been reduced-but we explained and asked them to stay for 14 or even 20 days. We issued medical certificates, did whatever we could.* (P5 - Municipality B)

In Municipality A, monitoring efforts focused on COVID-19-positive patients but extended to others in the community as well. Follow-up was done over the phone, and public guidance was shared through local radio and the city’s official website.

*We shared the daily bulletin on Facebook and Instagram. For families without [internet] access, we handed out printed flyers with prevention tips and safety instructions. People knew all the steps they had to take* [...]. (P1 - Municipality A)

According to participants from Municipality B, when patients with COVID-19 or other comorbidities experienced worsening symptoms, they contacted the PHU team. This occurred most often among residents of remote rural areas who lacked transportation. In such cases, the team used an ambulance or a municipal vehicle to travel to the patient’s home, either to provide care on-site or to transport them to the hospital. Although the roads connecting the municipality were paved, transportation remained challenging in some areas. Municipality A, in turn, had two ambulances-one larger and better equipped, and one smaller-as well as paved roads that facilitated access to neighboring towns.

*If seriously ill patients couldn’t come in, we went to get them. That’s just how it works in nonmetropolitan areas-and not only for COVID. Sometimes a patient calls, and if we have a vehicle available, the driver goes out to pick them up. We know there are people in rural areas who don’t have a car and can’t take the bus.* (P5 - Municipality B)

### Medications and vaccines: organization and distribution

In both municipalities, the validity of prescriptions for controlled medications was extended to six months, while prescriptions for other drugs-such as antihypertensives and antidiabetics-were valid for up to one year. In 2020 and 2021, routine follow-up visits, diagnostic tests referred to other municipalities, and both minor and major surgical procedures were suspended.

Participants from Municipality B reported that medications were dispensed at the PHU pharmacy. However, for patients with flu-like symptoms, a staff member would retrieve the medication and deliver it directly to them. This prevented symptomatic individuals from waiting in shared areas or coming into contact with other personnel outside the designated care zone. At times, certain medications were out of stock and were replaced with alternatives available at the PHU.

*There wasn’t a glass barrier at the pharmacy before, but during the pandemic they installed one to protect us. We had a lot of shortages. I think part of it was because of unnecessary treatments-many people were using antibiotics, dipyrone, fever reducers-and those ended up running out.* (P7 - Municipality B)

Similarly, participants stated that, when necessary, medications were prescribed according to the patient’s respiratory symptoms. They emphasized, however, that the term “COVID Kit” was not used with patients. In Municipality A, participants described distributing a “COVID Kit”, primarily to individuals who tested positive for COVID-19. This practice created problems, as some people believed that once they felt better, they could end their isolation early-or take the medications prophylactically, even without symptoms.

*We follow medication protocols based on the patient’s symptoms. Not everyone goes home with azithromycin, syrup, and dipyrone. Some leave with ibuprofen or an anti-inflammatory, but we prescribed based on symptoms. He* [a political figure] *wanted us to say we were using the “COVID Kit”, but in reality, it was just medication we already had at the PHU for treating symptoms.* (A1 - Municipality B)

*Then the “COVID Kit” arrived. We started using it, but before that, we just used regular cold medicine. Later, the kit seemed to help-it included antibiotics, vitamin D, and ivermectin.* (P5 - Municipality A)

The vaccination process was rolled out gradually, starting with priority groups based on age, healthcare workers, individuals with comorbidities, and bedridden patients. Participants from Municipality B reported high initial uptake; however, adverse reactions to the AstraZeneca vaccine discouraged some people from completing the full vaccination schedule. In response, the PHU team conducted active outreach, tracked users’ vaccination status, recorded doses, and implemented strategies to expand access-such as opening the unit on Saturdays. After receiving the vaccine, some residents felt comfortable returning to group activities like social gatherings in bars or religious services. As a result, the team had to reinforce public health guidance, emphasizing that despite the vaccine, social distancing was still necessary given the ongoing number of cases and deaths.

In Municipality A, once media coverage of the vaccine began, doses were administered according to the recommended priority groups. Initially, there was widespread hesitancy, as many believed the vaccine could cause harmful side effects. Over time, however, participants observed that symptoms were milder in vaccinated individuals, and as the death toll decreased, community uptake improved.

*Even when the vaccines arrived* [...] *many people were scared and didn’t want to take it. They thought they might die because there was still doubt about whether the vaccine was dangerous or not. But we know that what really brought down the number of COVID-19 cases in our municipality was the vaccination* [...]. (P2 - Municipality A)

## DISCUSSION

Based on this study’s findings, several investigations into the organization of PHC services during the COVID-19 pandemic highlighted the need to prioritize care for individuals with respiratory symptoms. These studies also emphasized the importance of training health professionals in emergency and urgent care, proper use of PPEs, and hand and environmental hygiene practices. These measures enabled the development of new care workflows within PHC services^(15,16)^.

It is important to recognize that rural settings present distinct challenges shaped by their populations’ social and productive ways of life. Historically, these areas have faced grassroots struggles, social disparities, and persistent health inequities. On a daily basis, rural populations are subject to individual, social, and systemic vulnerabilities^(1)^. According to Aliyi et al.^(17)^, rural residents tended to access information about COVID-19 through television or radio, lived near health units, and often had a history of chronic diseases or psychoactive substance use; in addition, they exhibited inadequate preventive practices, perceived a high risk of infection, and had conflicting understandings of the disease.

In this context, PHC responses to COVID-19 varied across rural municipalities, largely due to geographic barriers and limited service availability-since PHC was often the only healthcare option in these settings. Beyond the reorganizations already described, it was necessary to extend service hours, install tents outside PHUs, conduct home visits for those unable to access services, follow up on patients with flu-like symptoms, and engage community health workers in a wide range of activities, from monitoring to health education^(18-20)^.

In this scenario, the efforts of nursing teams to restructure their workflows to ensure population care stand out, particularly regarding the reorganization and management of services, with nurses serving as central figures. According to various studies^(21,22)^, professionals directly involved in the COVID-19 response reported increased workload, inadequate infrastructure, shortages of supplies and PPE, constant exposure to the virus, fear of infecting colleagues and family members, and a general lack of professional recognition. These factors contributed to the physical and mental strain experienced by healthcare workers. Such difficulties were even more pronounced in rural regions, where it was often challenging to retain staff over time due to high turnover. This fact compromised key attributes of PHC, especially continuity of care.

There was also a lack of health policies and system-level interventions to address these challenges in rural areas. The rural population was dispersed across remote locations, with living and working conditions that differed significantly from those in urban regions. In addition, health technologies remained concentrated in urban centers, making it difficult for professionals to refer patients to specialized care^(4)^.

A study conducted in China identified rural-urban disparities in knowledge, behavior, and mental health during the pandemic. Urban populations showed higher levels of understanding about the disease, accessed information primarily via the internet, and shared preventive strategies among themselves. In rural areas, however, information circulated mostly through community interaction. When symptoms appeared, people turned to health services. Anxiety and depression were more severe in urban populations than in rural ones^(23)^.

Because rural communities are often far from major urban centers, their risk of COVID-19 exposure was initially lower. However, once cases began to emerge, this population became more vulnerable due to limited healthcare resources^(24)^ and close-knit social habits such as neighborly contact and sharing. This scenario was further complicated by widespread misinformation and fake news, frequently amplified by Brazil’s then-president, Jair Messias Bolsonaro.

Despite these challenges, studies suggest that closer personal ties fostered stronger mutual care in municipalities with smaller populations. The community was seen as a key element in organizing health efforts in rural areas. Because rural populations are closely connected to their local context, their communities often had greater autonomy to manage localized strategies supported by PHC^(25)^. This appeared to be the case in Municipality B, where a stronger bond between residents and the PHC team contributed to greater adherence to mitigation strategies and preventing deaths.

In this context, social media played a critical role in disseminating information and promoting health education. Studies have shown that various communication channels were used, including social media platforms (WhatsApp, Instagram, Facebook), newspapers, radio, municipal websites, and telemedicine services^(26)^. However, in rural settings, internet connectivity was often unstable or lacked adequate coverage-conditions that proved especially critical during the pandemic^(27)^.

Another issue identified by participants-but still rarely addressed in the literature-was the fear of intubation due to COVID-19. A study involving older adults^(28)^ revealed that people experienced intense fear of dying from COVID-19 and of being intubated in hospital beds. This fear was primarily fueled by a lack of knowledge about the new disease and by the high number of deaths reported in Brazil and worldwide.

During the pandemic, using medications without scientific evidence became increasingly common. This practice, referred to as “early treatment” or the “COVID Kit”, aimed to treat or prevent infection. The drug combinations typically included hydroxychloroquine or chloroquine with azithromycin, ivermectin, and nitazoxanide, along with supplements like zinc and vitamins C and D. The use of these medications was associated with adverse effects and complications, such as cardiac problems, nausea, diarrhea, abdominal pain, drowsiness, dizziness, and itching^(29,30)^. Despite being discouraged by researchers, the “COVID Kit” took on a political dimension. Supporters of then president Jair Bolsonaro and his political allies publicly endorsed its use and, in some cases, pressured or coerced healthcare professionals into prescribing and distributing it.

According to participants in this study, that situation was observed in Municipality A, resulting in negative outcomes such as increased mortality rates, reduced adherence to social distancing, crowding, and a false sense of security regarding a cure. In addition, part of the population expressed fear of the COVID-19 vaccine when it became available in Brazil, believing it could be harmful. This hesitancy was also identified in other studies and was associated with misinformation, fake news, and the anti-vaccine movement-which found fertile ground in the country during that period^(31,32)^.

### Study limitations

The rural context is complex, shaped by multiple economic and political dynamics, which were reflected in the resistance and service reorganizations observed by PHC teams during the COVID-19 response. Because this period was marked by uncertainty and distress, many participants were unable to recall certain situations-possibly as a coping mechanism-which represents one of the study’s limitations. In addition, there is a need for further research in other rural areas, particularly in larger regions with predominantly rural populations, to better understand how these communities reorganized during the pandemic and how those changes may affect the post-COVID-19 context.

### Contributions to the Field

This study provides insight into the different ways in which COVID-19 was addressed and how these approaches influenced mortality rates during the pandemic. It also revealed that although PHC teams adopted similar strategies, the sociopolitical, cultural, and economic contexts varied considerably, resulting in disparities in the number of deaths between the two rural municipalities analyzed.

## FINAL CONSIDERATIONS

In light of these findings, it is possible to understand how COVID-19 was addressed through prevention, health promotion, adaptation, and the reorganization of PHC services in the two municipalities studied-each of which had a differing mortality rate. The nursing team, particularly nurses, carried out care coordination in both cases. Health directives, protocols, and technical guidelines were disseminated to the teams accordingly. However, in Municipality A, the “COVID Kit” was used to treat the disease, contrary to the available scientific evidence. When vaccines became available, the local population showed resistance to immunization, which may have contributed to the unfavorable outcome in that municipality. In Municipality B, extended community isolation, continuous population monitoring, and consistent support from the healthcare team throughout the pandemic may have contributed to the absence of deaths.

For the most part, the strategies adopted by the PHC teams in Municipalities A and B were similar. However, the populations demonstrated different behaviors and perceptions influenced by their sociocultural contexts. This understanding is grounded in health professionals’ perspectives, key stakeholders, and the researcher herself.

## Data Availability

The research data are available only upon request.
