# Supplementary material for: Nursing response and COVID-19 mortality in rural municipalities in southern Brazil
Source: Rev Bras Enferm. 2025 Nov 7;78(4):e20240332. doi: 10.1590/0034-7167-2024-0332 (PMC12594478; doi:10.1590/0034-7167-2024-0332)
Supplement: Translation

# Translation: Enfrentamento da enfermagem e a mortalidade por COVID-19 em municípios rurais do sul do Brasil

**DOI:** 10.1590/0034-7167-2024-0332pt

**Authors:** Lucia Helena Donini Souto, Janaína Barbieri, Nikole Martins de Figueiredo, Angela de Oliveira Carneiro, Adriano Maia dos Santos, Adriana Roese Ramos, Deise Lisboa Riquinho

**Affiliations:** I Universidade Federal do Rio Grande do Sul. Porto Alegre, Rio Grande do Sul, Brasil.; II Universidade Federal do Vale do São Francisco. Petrolina, Pernambuco, Brasil.; III Universidade Federal da Bahia. Salvador, Bahia, Brasil.

**Keywords:** Atenção Primária à, Saúde, Enfermagem, COVID-19, Mortalidade, Saúde da População Rural.

## Abstract

**Objetivos:**

compreender o enfrentamento da COVID-19 no âmbito da prevenção, promoção, adaptação e reorganização da Atenção Primária à Saúde em dois municípios rurais do sul do Brasil com diferentes taxas de mortalidade.

**Métodos:**

pesquisa qualitativa, tipo estudo de casos múltiplos, realizada por meio de entrevistas semiestruturadas com 20 participantes (profissionais da Atenção Primária à Saúde e atores-chave) e informações documentais. Emergiram três categorias: “Equipamentos, infraestrutura e acesso”; “Coordenação do cuidado pelos enfermeiros: monitoramento e isolamento” e “Medicamentos e vacinas: organização e distribuição”.

**Resultados:**

as equipes reorganizaram fluxos de atendimento, acompanhamento dos pacientes, aquisição de equipamentos e mudanças das salas para diminuir a transmissão viral. Os enfrentamentos da doença foram similares, porém divergências no âmbito político e no uso do “Kit COVID”.

**Considerações Finais:**

influências econômicas, políticas e culturais podem ocasionar diferentes taxas de mortalidade por COVID-19.

## INTRODUÇÃO

Nas localidades rurais, identificam-se desafios como o déficit de profissionais de saúde e a dificuldade de locomoção da população para a busca de serviços secundários e terciários devido a grandes distâncias e ausência de transportes. A pandemia de COVID-19 agravou esses problemas. Consequentemente, a Atenção Primária à Saúde (APS), como ordenadora e principal porta de entrada ao Sistema Único de Saúde (SUS), enfrentou adversidades (como resolução e encaminhamentos desse agravo em saúde) sobretudo em meio rural, onde é o único serviço disponível na maioria das vezes^(1,2)^.

Os casos e óbitos pela COVID-19 iniciaram nas regiões urbanas. Posteriormente, houve uma maior disseminação da doença em localidades rurais. Apesar disso, foram escassas as investigações que abordaram os impactos da pandemia nessas áreas.

Para realização plena de suas funções, os profissionais da Estratégia de Saúde da Família (ESF) atuantes em áreas rurais enfrentam dificuldades, como acesso geográfico, fatores econômicos e culturais, além de barreiras de informação. A equipe da ESF tem por característica identificar a singularidade e inserção sociocultural de cada usuário, considerando as especificidades do território. Essa atuação favorece a atenção integral, as ações de vigilância em saúde para proteção da saúde da população, prevenção, controle de riscos e agravos de doenças, bem como a promoção da saúde^(3,4)^.

No estado do Rio Grande do Sul (RS), observou-se que as aglomerações, principalmente urbanas, contribuíram para o aumento da transmissão da doença. As condições socioeconômicas também foram determinantes, seja pelo comércio exterior de produtos primários, seja pela presença de grandes concentrações de indústrias intensivas que demandam muita mão de obra, como é o caso dos frigoríficos. Estes foram os responsáveis por surtos da COVID-19 em cidades-polo, como Passo Fundo e Lajeado. Em decorrência da elevada concentração dos ramos da agroindústria e da mobilidade diária de seus trabalhadores, os entornos também foram afetados, com maior difusão da doença nos municípios de pequeno porte^(5,6)^.

No RS, ocorreu uma elevada disseminação da COVID-19 em municípios que tinham maior número de deslocamentos diários entre si e que possuíam uma maior densidade populacional: esses municípios apresentaram as maiores taxas brutas de mortalidade. Um estudo mostrou maior concentração de casos confirmados em municípios com algum tipo de carência urbana, como maior número de domicílios sem rendimentos, sem abastecimento de água, sem esgoto sanitário e com superlotação familiar^(7)^. Outro estudo associou partidarismo político ao aumento de óbitos em municípios, demonstrando que os municípios onde Jair Messias Bolsonaro - ex-presidente do Brasil e membro do Partido Liberal (PL) - teve mais votos em ambas as eleições (de 2018 e 2022) apresentaram um excesso de óbitos pela COVID-19 nos anos de 2020 e 2021^(8)^.

Pesquisas relacionadas a municípios rurais e à COVID-19 ainda são incipientes. Pouco se sabe sobre como a doença atingiu essas populações e quais foram as medidas tomadas por essas regiões para diminuir a transmissibilidade e impedir a contaminação das pessoas. As características das zonas rurais são específicas e requerem um aprofundamento para compreender tanto os diferentes fatores que desencadearam o aumento de óbitos pela COVID-19 quanto as estratégias tomadas para diminuir o contágio ou reduzir o número de óbitos^(3)^.

Diante do exposto, emergiu o seguinte questionamento: Como as equipes da APS enfrentaram a COVID-19 em municípios rurais com diferentes taxas de mortalidade?

## OBJETIVOS

Compreender o enfrentamento da COVID-19 no âmbito da prevenção, promoção, adaptação e reorganização da APS em dois municípios rurais no estado do Rio Grande do Sul, Brasil, com diferentes taxas de mortalidade.

## MÉTODOS

### Aspectos éticos

O estudo faz parte de uma pesquisa maior intitulada “Ambiente de trabalho e Saúde durante a pandemia de COVID-19: absenteísmo, *burnout*, gestão e organização do trabalho entre profissionais de enfermagem”, aprovada pelo Comitê de Ética em Pesquisa de um hospital público do estado do Rio Grande do Sul. Utilizou-se o Termo de Consentimento Livre e Esclarecido (TCLE) em todas as entrevistas. Para garantir o anonimato, empregou-se a letra “P” para profissionais e “A” para atores-chave, acompanhada de numeral cardinal representando a ordem das entrevistas e indicação do respectivo município (Município A ou Município B). Apenas os pesquisadores tiveram acesso ao material.

### Tipo de estudo

Trata-se de um estudo qualitativo descritivo, do tipo estudo de casos múltiplos. Ele permite que os investigadores focalizem um “caso” ou mais “casos”. Apesar das similaridades regionais, os dois casos analisados apresentaram diferentes taxas de mortalidade por COVID-19.

Este estudo é considerado uma estratégia investigativa dos fenômenos inseridos no contexto real, a qual possibilita apreender a totalidade das situações bem como descrever, compreender e interpretar a complexidade de cada caso, podendo os casos ser comparáveis. O manuscrito foi preparado de acordo com a lista de verificação de critérios COREQ^(9,10)^.

### Cenário do estudo

O estudo foi realizado em dois municípios rurais do estado do Rio Grande do Sul com diferentes taxas de mortalidade por COVID-19. De 2020 a 2021, o Município A tinha a segunda maior taxa de mortalidade acumulativa por 100 mil habitantes (661), ao passo que o Município B apresentava a menor taxa (0)^(11)^. A escolha por esses municípios deu-se por serem de regiões de saúde próximas (não identificadas para manter o anonimato da pesquisa), terem majoritariamente população rural (71% cada um)^(12)^ e apresentarem UBS com equipes de Saúde da Família (eSF) e multidisciplinares (eMulti).

Quanto a características sociodemográficas, o Município A apresentava uma comunidade indígena, parte da população trabalhava em frigoríficos, e o partido político do prefeito naquele período era o Partido Progressista (PP). Apesar de terem sido relatados casos de COVID-19 entre indígenas, não houve óbitos. No Município B, havia maior concentração de pequenos agricultores, população idosa, e o prefeito era do mesmo partido: PP.

### Fonte de dados

Nesses locais, foram convidados todos os profissionais de saúde (18 do Município A e 23 do Município B) e atores-chave (secretários de saúde e representantes regionais das Coordenadorias de Saúde, seis possíveis participantes). Considerou-se como critério de inclusão a atuação de no mínimo seis meses antes do início da pandemia. Já os de exclusão foram os afastamentos por motivo de saúde e/ou férias no período da geração dos dados. Três profissionais do Município A encontravam-se em férias nesse período.

Participaram 16 profissionais da APS (9 do Município A e 7 do B) e 4 atores-chave (1 do Município A e 3 do B), totalizando 20 participantes. Foram selecionados de maneira intencional, buscando representar as diferentes categorias profissionais. Embora houvesse outras profissões que preenchiam os critérios, os profissionais não se dispuseram a participar. Um ator-chave do Município A, inicialmente eleito para entrevista, cancelou-a. Os depoimentos de todos os participantes foram considerados para compor as categorias analíticas.

O tamanho da amostra foi orientado pelo princípio do “poder da informação”, considerando o objetivo do estudo, especificidade da amostra, teoria, qualidade do diálogo e estratégia de análise^(13)^. Os profissionais nos dois municípios foram quatro enfermeiros(as), quatro técnicos(as) de enfermagem, seis agentes comunitários(as) de saúde, um(a) psicólogo(a), um(a) farmacêutico(a). Os atores-chave eram enfermeiros(as) e administrador(a).

### Coleta e organização dos dados

Antes de ir aos municípios, três enfermeiras pesquisadoras (uma doutora, uma mestre e outra especialista) com experiência em pesquisa qualitativa reuniram-se com os atores-chave por videoconferência e apresentaram o estudo, combinando a data da visita e posteriormente as entrevistas. As entrevistas semiestruturadas foram realizadas pela enfermeira especialista de junho a julho de 2023. Elas ocorreram de forma individual, conforme a disponibilidade de cada participante, durante suas jornadas de trabalho, em uma sala reservada na UBS, na secretaria de saúde ou, no caso dos representantes das coordenadorias de saúde, por videoconferência em plataforma virtual. Todas foram gravadas em áudio e posteriormente transcritas, com duração aproximada de 40 minutos.

Além das entrevistas semiestruturadas, informações sobre o contexto estudado foram obtidas de documentos, como os sites dos municípios, notícias publicadas e registros em arquivos disponibilizados pelos municípios (atas das reuniões realizadas naquele período, ofícios, decretos e portarias municipais). O uso de várias fontes de evidência torna-se importante para a garantia de confiabilidade do estudo de casos múltiplos. Tal procedimento denomina-se “triangulação dos dados” e visa construir um encadeamento de evidências, aumentando a acurácia das informações e interpretações.

### Análise dos dados

O material das entrevistas foi submetido à análise de conteúdo do tipo temática^(14)^, realizada seguindo a recomendação das três etapas: pré-análise; exploração do material; e tratamento dos resultados obtidos e interpretação. As análises foram feitas com base nas boas práticas relacionadas à COVID 19 e foram interpretadas à luz da literatura e contexto investigado. Para organizar e apoiar a formulação das categorias, utilizou-se como ferramenta o software Nvivo versão 14.1.

Ao final, realizou-se a análise do estudo de casos múltiplos por meio de uma explicação narrativa. Explicar um fenômeno significa estipular um conjunto de elos causais em relação a ele, ou seja, dizer “como”” ou “por que” algo aconteceu.

Assim, três categorias (apresentadas na seção Resultados) responderam à questão norteadora: “Equipamentos, infraestrutura e acesso”; “Coordenação do cuidado pelos enfermeiros: monitoramento e isolamento” e “Medicamentos e vacinas: organização e distribuição”.

## RESULTADOS

### Equipamentos, infraestrutura e acesso

No Município A, foi separado um espaço para atender os pacientes sintomáticos respiratórios. Além disso, ocorreram visitas domiciliares, e a UBS permaneceu aberta 24 horas por dia. No plantão, duas pessoas ficavam de sobreaviso caso algum paciente solicitasse atendimento. Inicialmente havia poucos EPIs, porém, após os primeiros meses, já não havia problemas com abastecimento. Os participantes relataram que foi necessário montar uma sala equipada para atendimentos de quadros graves, caso os pacientes não conseguissem leitos no hospital de referência.

Os participantes do Município B modificaram a utilização das salas na UBS para atender de forma isolada os usuários com sintomas respiratórios, desde a entrada no consultório até a saída da unidade. A estrutura física limitada e os consultórios pequenos acabavam por expor toda a equipe, apesar da presença dos EPIs.

*A gente procurou fazer uma sala específica para o atendimento, onde os pacientes com sintomas não tivessem tanto contato com os outros pacientes* [...]. (A1 - Município B)

*Demorou um pouco para vir. Teve momentos que tínhamos e outros não* [...]. *Todo mundo sofreu com essa questão dos EPIs. Às vezes, tinha apenas aquela quantidade - logo no início que todo mundo está querendo.* (P1 - Município A)

No Município B, foi criado um fluxo diferente para atender os pacientes com sintomas respiratórios: protocolo de medicações, três profissionais escalados para o atendimento, testagem dos pacientes com sintomas gripais e monitoramento daqueles que positivaram para COVID-19. Diariamente eram feitas ligações aos pacientes, sobretudo aos que apresentavam sintomas mais graves como febre. Se necessário, realizava-se visita domiciliar, verificavam-se os sinais vitais, e encaminhava-se ao hospital. As orientações contidas nas notas técnicas do governo do estado eram repassadas pela Coordenadoria Regional de Saúde. Na perspectiva dos participantes, essa ação contribuiu para que o município não apresentasse nenhum óbito.

Em ambos os municípios, diminuiu-se o fluxo de atendimento usual na UBS: o foco eram os atendimentos para pessoas com sintomas respiratórios, urgências e emergências. Um ator-chave do Município B relatou que, para reduzir o movimento no serviço de saúde, eram feitas visitas domiciliares para realização de testagem, administração de medicamentos e cuidado com os pacientes com COVID-19. O monitoramento dos pacientes era realizado pela equipe de enfermagem.

*Eu acredito que isso foi um ponto bem importante, ter esse controle maior, e aqui a gente conseguia ligar e conseguia monitorar bem* [...]. (A1 - Município B)

*Esse monitoramento que a equipe de enfermagem realizava* [...] *usavam uma planilha, sabiam quantos estavam com a COVID-19, quantos estavam em isolamento e como era o quadro de cada paciente.* (P1 - Município A)

### Coordenação do cuidado pelos enfermeiros: monitoramento e isolamento

Os participantes do Município A relataram que a gestão do serviço era realizada pelos enfermeiros e que estes participaram de capacitações e treinamentos, os quais já ocorriam; porém, naquele momento, a prioridade eram os atendimentos graves para os pacientes com sintomas respiratórios e risco de parada cardiorrespiratória, bem como a paramentação. Portanto, eles se prepararam, principalmente, para atender esses casos.

Da mesma forma, no Município B, os participantes relataram que os enfermeiros coordenaram os atendimentos prestados durante a pandemia. Assim, a triagem era feita na entrada da unidade, priorizando os atendimentos para síndromes gripais e casos de emergência e/ou urgência. Por vezes, foi necessário estender o horário de atendimento em razão da alta demanda. Eles entendiam que era necessária uma maior valorização da categoria de enfermeiros e técnicos de enfermagem, principalmente quanto a questões salariais como o piso da enfermagem, que ainda não tinha sido aprovado na época.

*A enfermeira sempre orientou o que a gente tinha que fazer, organizar as visitas domiciliares, mas sempre com cuidado tanto para as pessoas quanto para a equipe, em razão do risco de contaminação, porque há nossas famílias também, então tinha que ter cuidado.* (P2 - Município A)

As visitas domiciliares foram modificadas: preferencialmente, eram realizadas na parte externa das residências. Os grupos para idosos, pessoas com doenças crônicas não transmissíveis (DCNT) e gestantes foram suspensos, pois não deviam ocorrer aglomerações.

*A gente não entrava na casa da família, permanecia do lado de fora na parte externa, onde havia circulação de ar e maior ventilação.* (P2 - Município B)

*A gente realizava visita domiciliar diariamente, todos os dias; e, se o paciente necessitasse, ligaria para UBS, que tinha equipe disponível 24 horas. A gente ficava tipo de sobreaviso. Tem o turno da noite aqui, né, mas tinha o plantão em que ficava um técnico de enfermagem e um enfermeiro.* (P6 - Município A)

No Município A, as visitas domiciliares eram feitas aos pacientes com a doença, em que se avaliavam os sinais vitais e se fazia o acompanhamento diariamente. Se necessário, levava-se a equipe de enfermagem e médica junto, pois muitos pacientes não queriam ir ao hospital; logo, toda a equipe fazia essa busca, orientava e tentava fazer com que os pacientes procurassem a UBS, principalmente quando o estado de saúde era mais grave, com risco de óbito. Todavia, os participantes informaram que os usuários demoravam muito tempo para ir à UBS ou não queriam por medo da intubação e de morrer no hospital.

*Eu fiz uma visita, e o usuário estava grave. Quando eu cheguei no local, realmente estava mal. Eu orientava a ir para UBS para realizar uma medicação e, se necessário, ir para o hospital, mas o paciente dizia que não queria ir* [...]. *Daí eu conversava com o médico para ir e tentar convencer o usuário a vir para a UBS* [...]. (P7 - Município A)

No Município B, os participantes relataram que os pacientes com a doença permaneceram em isolamento por 15 dias. Após alguns meses, as notas técnicas começaram a instruir a diminuição dos dias; porém, eles informaram que, mesmo assim, mantinham por mais tempo os pacientes em isolamento e forneciam atestado se necessário. Entende-se que isso também foi uma forma de diminuir a transmissão. Além disso, eles monitoravam os pacientes com sintomas respiratórios, usando diferentes recursos para orientar a população, como teleconsulta e divulgação nas redes sociais, na rádio e no site do município.

*Olha, na verdade, a gente segurou tudo o que podia, até a questão dos isolamentos. Levou um tempo, porque, quando o Ministério da Saúde resolveu diminuir os isolamentos de 14 dias para 10 dias. No entanto, nós levamos mais tempo isolando por 14 dias. As pessoas questionavam o porquê, já que estava liberado para 10 dias, mas a gente explicava e pedia para que ficassem 14 ou 20 dias. Fornecia atestado, enfim, fazia o possível.* (P5 - Município B)

No Município A, focalizou-se a questão do monitoramento dos pacientes, tanto os que positivaram para COVID-19 quanto os demais. Assim, o acompanhamento era feito por telefone, e as orientações à população eram repassadas pela rádio e no site do município.

*Um jornal era passado pelo Facebook e Instagram. Para as famílias que não tinham acesso* [a esses meios]*, era distribuído folder como forma de prevenção, com os cuidados que se tinha que ter e tudo mais. As famílias sabiam de todos os cuidados* [...]. (P1 - Município A)

Conforme o relato dos participantes do Município B, quando os pacientes apresentavam alguma gravidade relacionada à COVID-19 ou a outras comorbidades, eles contatavam a equipe da UBS. Assim, principalmente no caso daqueles que moravam em regiões mais afastadas do centro da cidade e que não possuíam meios de locomoção, a equipe da UBS deslocava-se de ambulância ou com carro da Prefeitura até a residência a fim de buscá-los para atendimento ou, se necessário, encaminhá-los ao hospital. Ademais, as estradas que interligam o município eram asfaltadas, dificultando o transporte. Já o Município A dispunha de duas ambulâncias, uma maior e mais equipada e outra menor; e havia estradas asfaltadas para interligar o município aos seus vizinhos, facilitando o percurso.

*Os pacientes graves, se eles não conseguiam vir, a gente ia* [buscar]*. A questão do interior, a gente tem uma coisa que funciona, não só para COVID-19. Às vezes, o paciente liga, e se a gente tem um carro disponível, o motorista vai e busca o paciente, porque a gente sabe que tem pacientes que moram no interior, que não têm condições, não têm carro e não conseguem vir de ônibus.* (P5 - Município B)

### Medicamentos e vacinas: organização e distribuição

Nos dois municípios, a validade das receitas medicamentosas foi estendida para seis meses para medicações de controle especial, enquanto os demais medicamentos (como anti-hipertensivos e antidiabéticos) passaram a valer por um ano. As consultas eletivas de acompanhamento, os exames realizados em outros municípios e as pequenas ou grandes cirurgias foram suspensos em 2020 e 2021.

Os participantes do Município B informaram que os medicamentos eram dispensados na farmácia, mas para os pacientes com algum sintoma gripal, um profissional retirava a medicação e entregava a eles. Assim, os pacientes sintomáticos respiratórios não permaneciam esperando os medicamentos nem entravam em contato com nenhum outro profissional que não estivesse naquele setor específico. Havia períodos em que faltavam alguns medicamentos, os quais eram substituídos pelos disponíveis na UBS.

*Antes não havia uma barreira de vidro na farmácia, mas com a pandemia eles* [a] *colocaram como uma proteção para nós. Teve bastante falta de medicamento. Eu acho que, às vezes, era em virtude do tratamento desnecessário, porque muitos utilizavam antibiótico ou dipirona, antitérmico, e essas coisas acabaram faltando.* (P7 - Município B)

Da mesma forma, foi relatado que, se necessário, eram prescritos medicamentos aos pacientes de acordo com os sintomas respiratórios apresentados. Todavia, os participantes sinalizaram que o termo “Kit COVID” não era usado com os pacientes. No Município A, eles relataram que havia o “Kit COVID”, entregue principalmente àqueles que positivaram para COVID-19. Perceberam que isso se tornou um problema, pois as pessoas entendiam que, estando melhor dos sintomas, poderiam sair do isolamento ou tomar as medicações sem apresentar nenhum sintoma, como uma forma de profilaxia.

*A gente segue algumas medicações conforme os sintomas do paciente. Nem todos os pacientes vão sair com azitromicina, xarope e dipirona. Alguns vão sair com um ibuprofeno, digamos, um anti-inflamatório. No entanto, a gente medicava as pessoas conforme os sintomas. Mas ele* [líder político] *queria que nós disséssemos que a gente usava o “Kit COVID”. No entanto, eram medicações da UBS que a gente tinha por causa dos sintomas.* (A1 - Município B)

*Veio logo o “Kit COVID”. No caso, a gente começou a tratar, mas antes não tinha o tratamento: tratava-se com remédios normais de gripe, mas depois foi dando resultado com o “Kit COVID”, que ele vem com antibiótico, vitamina D e ivermectina.* (P5 - Município A)

Já o processo de vacinação ocorreu de maneira gradual: inicialmente para grupos prioritários conforme faixa-etária, profissionais de saúde, pessoas com comorbidades e acamados. Os participantes do Município B informaram que a procura para tomar a vacina foi grande na época; porém, a vacina do laboratório AstraZeneca causava reações adversas, consequentemente os pacientes não tomavam as demais doses. Assim, realizava-se busca ativa, monitorava-se a situação vacinal dos usuários, registravam-se as doses aplicadas e adotavam-se estratégias para ampliar o acesso da população, como a abertura da unidade aos sábados. Com a vacinação, algumas pessoas se sentiam autorizadas a voltar para suas atividades coletivas, como aglomerações em bares ou igrejas. Logo, a equipe precisou orientar que o momento ainda não permitia isso e que era necessário manter o distanciamento social, pois havia muitos casos e óbitos.

No Município A, assim que a mídia veiculou a notícia sobre a vacinação, começou a aplicação das doses conforme os grupos prioritários. Inicialmente, houve resistência por parte da população, pois muitos acreditavam que a vacina causaria efeitos adversos. Posteriormente, foram percebendo que os sintomas eram mais leves naqueles que haviam se vacinado; e, com a diminuição dos óbitos, aderiram mais à vacinação.

*Mesmo quando chegou as vacinas* [...] *muitos tinham medo, não querendo fazer, achando que iam morrer, porque ficou aquela dúvida se a vacina era perigosa ou não. No entanto, a gente sabe que a COVID-19 diminuiu no nosso município foi depois da vacinação mesmo* [...]. (P2 - Município A)

## DISCUSSÃO

De acordo com os resultados da pesquisa, diversos estudos sobre a organização dos serviços da APS mostraram que foi necessário priorizar os atendimentos das pessoas com sintomas respiratórios durante a pandemia de COVID-19. Também se destacou a importância da capacitação dos profissionais para atendimentos de urgência e emergência, uso adequado de EPIs e higienização das mãos e do ambiente de trabalho. Essas medidas possibilitaram a definição de um novo fluxo de atendimento aos usuários^(15,16)^.

No entanto, vale salientar que o contexto rural é um cenário específico em virtude dos modos de viver e produzir de seus povos. Há historicamente embates populares, disparidades sociais e iniquidades em saúde. Cotidianamente, as populações rurais enfrentam vulnerabilidades individuais, sociais e programáticas^(1)^. Conforme Aliyi *et al.* ^(17)^, os moradores rurais obtinham informações sobre a doença por meio de TV ou rádio, residiam perto de Unidades de Saúde, apresentavam histórico de doenças crônicas e uso de substâncias psicoativas; além disso, demonstravam práticas inadequadas contra a COVID-19, percepção de alto risco de contaminação e entendimento controverso sobre a doença.

Dessa forma, houve diferenças dos cuidados da APS para o enfrentamento da COVID-19 em municípios rurais, seja pela dificuldade de acesso geográfico, seja pela limitação de oferta, já que a APS era o único serviço disponível na maioria dos cenários. Assim, além das reorganizações descritas, foi necessário ampliação do horário de atendimento, montagem de toldos na parte externa das UBS, visita domiciliar para quem não conseguia acessar o serviço, acompanhamento domiciliar dos casos de síndromes gripais e atuação dos agentes comunitários de saúde em diversas atividades, desde o monitoramento até as orientações em saúde^(18-20)^.

Nesse sentido, importa destacar o esforço da equipe de enfermagem para modificar seus processos de trabalho em prol do cuidado da população, principalmente na reorganização e gestão desses serviços tendo as(os) enfermeiras(os) como protagonistas. Conforme diferentes estudos^(21,22)^, os profissionais diretamente envolvidos e atuantes no enfrentamento da COVID-19 apresentaram aumento na sobrecarga de trabalho, relatando falta de estruturas adequadas, escassez de materiais e EPIs, contato direto com a doença, medo da contaminação e transmissão aos colegas e familiares, além da falta de valorização profissional. Tais fatores ocasionaram comprometimento da saúde física e mental dos trabalhadores. Esses problemas se intensificaram ainda mais em regiões rurais, principalmente pela dificuldade de fixar profissionais por longos períodos e pela alta rotatividade, prejudicando atributos da APS como a longitudinalidade em saúde.

Também houve carência de políticas e intervenções do sistema de saúde que pudessem contribuir para sanar tais dificuldades nessas áreas. A população rural encontrava-se dispersa em áreas de difícil acesso, com condições de vida e trabalho distintas das regiões urbanas; além do mais, as tecnologias permaneceram centradas nessas localidades, sendo um desafio aos profissionais de saúde encaminhar os usuários para tratamentos especializados^(4)^.

Em um artigo desenvolvido na China, identificaram-se disparidades rurais-urbanas em conhecimento, comportamento e saúde mental no período pandêmico. As populações urbanas apresentaram níveis de conhecimento sobre a doença maiores do que as populações rurais. O acesso às informações se dava pela internet, e as medidas preventivas eram compartilhadas entre as pessoas. Já em áreas rurais, as informações circulavam sobretudo por meio da interação comunitária; quando apresentavam sintomas da doença, as pessoas recorriam aos serviços de saúde. Os quadros de ansiedade e depressão foram mais graves em populações urbanas do que em populações rurais^(23)^.

Por serem afastadas dos grandes centros urbanos, as regiões rurais contavam com menor probabilidade de contaminação pela COVID-19; porém, quando os casos começaram a surgir, houve uma maior vulnerabilidade dessa população devido à escassez de recursos de saúde^(24)^ e aos hábitos de vizinhança e partilha. Esse cenário foi agravado pelo contexto de desinformação e *fake news*, amplamente disseminadas pela figura do então presidente do Brasil, Jair Messias Bolsonaro.

Apesar disso, estudos indicam que, em municípios com menor número de habitantes, os vínculos mais estreitos entre as pessoas favorecem um maior cuidado mútuo. Entende-se que a comunidade é um elemento-chave na organização dos cuidados em localidades rurais. As populações rurais conhecem o contexto local, portanto suas comunidades têm uma maior autonomia para gerenciar ações específicas nos territórios, com o apoio da APS^(25)^. Este parece ter sido o caso no Município B, onde havia vínculo maior entre a população e a equipe da APS e maior adesão às estratégias para diminuir a transmissibilidade da COVID-19 e evitar óbitos.

Nessa conjuntura, as mídias sociais foram determinantes para a divulgação de informações e educação em saúde. Estudos mostraram diferentes meios utilizados: redes sociais (WhatsApp, Instagram, Facebook), jornais, rádios, sites dos municípios e telemedicina^(26)^. Todavia, no ambiente rural, sabe-se que a maioria das conexões de internet precisa de melhorias na estabilidade e na cobertura, o que justamente se mostrou essencial durante a pandemia^(27)^.

Outra questão identificada pelos participantes do presente estudo, mas ainda pouco abordada, foi sobre o medo da intubação devido à COVID-19. Um estudo com idosos^(28)^ evidenciou que as pessoas tinham excessivo medo da morte por COVID-19 e da intubação em leitos dos hospitais, especialmente pela falta de conhecimento sobre a nova doença e pelo grande número de óbitos ocorridos no Brasil e no mundo.

Já em relação ao uso de medicações, notou-se um aumento durante a pandemia. Nesse sentido, a combinação de medicamentos sem evidências científicas ganhou espaço, senda denominada de “tratamento precoce” ou “Kit COVID”, visando tratar ou prevenir a doença. O combo de medicamentos usados para essa finalidade foi a hidroxicloroquina ou cloroquina junto com a azitromicina, ivermectina e nitazoxanida, além dos suplementos de zinco e vitaminas C e D. Esse consumo associou-se a possíveis reações adversas e complicações, como problemas cardíacos, náuseas, diarreias, dores abdominais, sonolências, tonturas e pruridos^(29,30)^. Mesmo sendo desaconselhado por estudiosos, tais medicações tiveram uma conotação político-partidária, em que apoiadores do então presidente (Jair Bolsonaro) e correligionários adquiriram os tais “Kit COVID”, manifestaram-se publicamente favoráveis ao uso e coagiram ou constrangeram profissionais de saúde à prescrição e distribuição.

Conforme a perspectiva dos participantes da pesquisa, essa situação foi observada no Município A, trazendo desfechos desfavoráveis como o aumento da taxa de mortalidade, diminuição do isolamento social, aglomerações e falsa ideia de cura da doença. Ademais, parte da população demonstrou receio em relação à vacinação contra a COVID-19 quando o imunizante chegou ao Brasil, por acreditar que poderia causar algum malefício. Essa hesitação também foi apontada em outros estudos, associada à desinformação, *fake news* e movimento antivacina, que encontrou solo fértil no país, naquele período^(31,32)^.

### Limitações do estudo

O contexto rural mostra-se complexo, marcado por múltiplas interações econômicas e políticas, refletidas nas resistências e reorganizações observadas pelas equipes da APS durante o enfrentamento da COVID-19. Por ser um período que ocasionou apreensão, muitos participantes não lembravam de determinadas situações, possivelmente como forma de lidar com o ocorrido, o que representa uma das limitações do estudo. Além disso, verifica-se a necessidade de novas pesquisas em outras áreas rurais, especialmente em regiões mais extensas com predominância de população rural, a fim de compreender suas formas de reorganização e as implicações no período pós-pandemia de COVID-19.

### Contribuições para a Área

Este estudo permite compreender as diferentes formas de enfrentamento da COVID-19 e o modo pelo qual elas influenciaram as taxas de mortalidade naquele período. Este trabalho também mostrou que as estratégias adotadas pelas equipes da APS foram similares, porém os contextos sociopolíticos, culturais e econômicos foram divergentes, gerando disparidades no número de óbitos entre os dois municípios rurais estudados.

## CONSIDERAÇÕES FINAIS

Diante do exposto, pode-se compreender o enfrentamento da COVID-19 no âmbito da prevenção, promoção, adaptação e reorganização da APS nos dois municípios estudados, considerando suas diferentes taxas de mortalidade. A coordenação do cuidado em saúde em ambos foi realizada pela equipe de enfermagem, principalmente pelas(os) enfermeiras(os). Nesse sentido, os fluxos, os direcionamentos bem como as normativas e notas técnicas foram encaminhados para as equipes. Todavia, no Município A, o “Kit COVID” foi utilizado como tratamento para a doença, em desacordo com as evidências científicas disponíveis. Com o surgimento do imunizante, essa população apresentou resistência para se vacinar, o que pode ter contribuído para o desfecho desfavorável nesse município. No Município B, o isolamento prolongado da comunidade, o monitoramento contínuo da população e o apoio da equipe para continuidade do cuidado e enfrentamento da pandemia podem ter contribuído para a ausência de óbito.

Em sua maioria, as estratégias adotadas pelas equipes de saúde dos municípios A e B foram semelhantes no enfrentamento da doença. No entanto, as populações apresentaram comportamentos e percepções diferentes, influenciadas por seus contextos socioculturais. Essa compreensão baseia-se nas perspectivas dos profissionais de saúde, atores-chave e da própria autora do estudo.

## Data Availability

Os dados de pesquisa só estão disponíveis mediante solicitação.
